# How team leader narcissism influences team task performance: the mechanisms of team identification, team member narcissism and task initiative

**DOI:** 10.3389/fpsyg.2026.1730577

**Published:** 2026-03-02

**Authors:** Yingzi Liu, Huanzhen Liu, Xiaokang Wang, Heetae Park

**Affiliations:** 1 School of Business Administration, Dong-A University, Busan, Republic of Korea; 2 Shandong College of Economics and Business, Weifang, China

**Keywords:** social identity theory, team identification, team leader narcissism, team member narcissism, team task performance, team member task initiative

## Abstract

**Introduction:**

In contemporary organizational contexts, leader narcissism has received increased scholarly attention because of its complex impact on team effectiveness. Given the critical role of team level performance, understanding the mechanisms by which leader narcissism influences team outcomes is of paramount importance. From the perspective of social identity theory, we examined the mediating effect of team identification and moderating roles of member narcissism and task initiative.

**Methods:**

Survey data were collected from 640 employees and their leaders across 160 teams in China.

**Results:**

The primary findings are as follows. First, leader narcissism negatively impacted team task performance. Second, team identification played a significant mediating role in the relationship between leader narcissism and team task performance. Third, member narcissism moderated the leader narcissism and team identification relationship, such that higher member narcissism intensified this negative association. While team member task initiative enhances the positive impact of team member team identification on team task performance, the effect may instead exacerbate the risk of performance decline in leader narcissism contexts.

## 1 Introduction

As organizational environments become increasingly complex and uncertain, traditional hierarchical structures have fundamentally shifted toward more collaborative, team-based configurations. This transition is not only about enhancing agility and internal coordination (Kozlowski and Bell, 2013; Mathieu et al., 2017) but is also driven by the recognition that complex, multifaceted problems often exceed the capabilities of individual contributors, necessitating the integration of diverse knowledge, skills, and perspectives. Within this shift, team leaders not only manage task distribution and resource coordination but also significantly contribute to shaping the team climate, fostering collective identification, and maintaining internal cohesion (Friedrich et al., 2009; Van der Voet and Steijn, 2021; Zaccaro et al., 2001). In this context, leaders’ individual traits, such as narcissism, have gained importance because they significantly influence team dynamics and collaborative processes.

As leadership research increasingly emphasizes leader traits and individual differences, narcissism is attracting growing attention because it shapes how leaders relate to others and pursue status and recognition (Antonakis et al., 2012; Zaccaro et al., 2018). This study focuses on grandiose narcissism—a relatively stable trait characterized by exaggerated self-importance, a chronic need for admiration, and a tendency to dominate social interactions to maintain a positive self-image (Back et al., 2013; Campbell et al., 2011). Although narcissistic leaders can sometimes appear confident and charismatic, grandiose narcissism is also associated with self-serving and exploitative tendencies, such as credit claiming, resource manipulation, and reduced accountability. These are particularly harmful in task-interdependent teams that rely on collaboration and shared commitment (Badar et al., 2023; Choi and Phan, 2022). This focus aligns with our conceptualization and measurement of leader narcissism as subclinical grandiose narcissism, foregrounding its antagonistic and self-serving implications for team functioning.

Against this backdrop, at least two critical gaps can be identified in the literature on leader narcissism. First, although research on the individual-level attitudinal and behavioral consequences of narcissism is extensive, its dynamics at the team level remain underexplored. Only a few studies have examined how the narcissistic composition of teams affects performance and its evolution over time (Felty et al., 2015; Grijalva et al., 2020). Additionally, integrative, team-level tests linking leader narcissism to team-level psychological states and subsequently to team task performance remain limited (Braun, 2017; Schmid et al., 2021). This gap matters because narcissistic leader behaviors are enacted and interpreted in teams, with consequences that can extend to the team level (Braun, 2017). In such interactional contexts, shared experiences and perceptions, as well as other emergent states develop through team interaction and influence team effectiveness (Fyhn et al., 2023; Mathieu et al., 2019). Accordingly, individual-level evidence alone is not sufficient to directly test whether and how leader narcissism affects team task performance via team-level psychological mechanisms (Fodor et al., 2021). Second, the mediating and moderating mechanisms through which leader narcissism influences team and follower outcomes remain unclear, particularly with respect to the roles of team members’ characteristics and task-related behaviors (Braun, 2017; Schmid et al., 2021). In other words, existing evidence is fragmented, and it is not clear which combinations of follower compositions and behavioral patterns systematically amplify or attenuate the negative effects of leader narcissism (Den Hartog et al., 2020; London, 2019). Recent work suggests that follower behaviors and traits can meaningfully shape how leader narcissism plays out, underscoring the need to specify the follower compositions and behavioral patterns that strengthen or weaken these effects (Den Hartog et al., 2020; Nevicka et al., 2018). These boundary conditions are directly relevant to how teams are staffed and composed. Team composition shapes the emergence of affective, behavioral, and cognitive states that influence whether teams meet their objectives (Bell et al., 2018), and integrative work clarifies how different composition models connect member attributes to team outcomes (Mathieu et al., 2014). Diverging from prior work that predominantly examined how narcissism shapes employees’ attitudes and behaviors at the individual level, the present study adopts a multilevel, contextual perspective and develops a team-level model that examines how leader narcissism and team compositional characteristics are related to team identification and team task performance, focusing on between-team differences.

To address these gaps, this study draws on social identity theory (SIT) as an overarching theoretical framework and focuses on team identification as a core team-level psychological mechanism. SIT posits that individuals define “who they are” partly through membership in social groups. Social identity constitutes the aspect of the self-concept derived from emotional attachment to and value-based identification with a group (Ashforth and Mael, 1989; Tajfel and Turner, 1979). When members incorporate the team into their self-definition, they are more inclined to protect the team’s positive image and to behave in line with collective norms and goals, thereby enhancing their sense of belonging, cooperation, and performance (Hogg et al., 2012). In team settings, leaders are often seen as prototypical representatives of the group, and their behavior critically shapes whether the team is perceived as a worthy target of identification or not (Van Knippenberg, 2011). Supportive and fair leadership tends to strengthen team identification and promote voluntary effort and collaboration, whereas self-centered, power-abusing, or unfair leadership is likely to weaken team identification, trigger psychological withdrawal, and impair performance (Fransen et al., 2015; Van Dijk and Dick, 2009). Narcissistic leaders who claim disproportionate credit, ignore distributive and procedural fairness, and excessively pursue personal prestige convey a clear signal that their personal goals take precedence over team interests (Choi and Phan, 2022; Rosenthal and Pittinsky, 2006). From a social identity perspective, such perceptions constitute a threat to the team’s identity value, reduce members’ willingness to incorporate the team into their self-concept, and diminish team identification, which in turn undermines team task performance. Accordingly, team identification can be viewed as a key team-level mediating mechanism linking leader narcissism to team task performance.

Moreover, the consequences of leader narcissism hinge on who the followers are and how they engage in task behavior. Accordingly, we examine member narcissism and member task initiative as two theoretically grounded boundary conditions that shape how leader narcissism is interpreted in teams and how team identification translates into task performance. Within the broader framework of grandiose narcissism, this study distinguishes between two related but conceptually distinct forms of narcissism from a role-based perspective: leader narcissism, referring to the grandiose narcissism of the formal team leader, and member narcissism, referring to the grandiose narcissism of individuals team members. The former concerns who represents the team and holds formal authority, whereas the latter reflects the team’s member composition. In this study, member narcissism is conceptualized as a team compositional characteristic.

From a social identity perspective, leader behaviors undermine team identification to the extent that they are interpreted as identity-relevant cues that the leader is prioritizing personal status over collective interests and is thus failing to function as a prototypical and fair in-group representative (Van Knippenberg, 2011). Such interpretations should be particularly pronounced when the team is composed of members who are highly attuned to status and external validation (e.g., recognition and admiration), as emphasized in the grandiose narcissism literature (Grapsas et al., 2020; Morf and Rhodewalt, 2001). Accordingly, we examine member narcissism as a team compositional boundary condition. When member narcissism is high, members may be especially attuned to status-relevant cues in leaders’ behaviors and thus are more likely to interpret credit-claiming and self-promotion as self-interested status pursuits that disadvantages others. This, in turn, can intensify identification-related damage in teams (Liu et al., 2022; Nevicka and Sedikides, 2021). Recent work on leader-follower narcissism suggests that when narcissistic traits are present in both leaders and followers, their interactive effects can be quite complex (Liu et al., 2025). However, empirical evidence is still lacking on how the overall level of member narcissism shapes the team-level consequences of leader narcissism.

Task initiative reflects proactive, future-oriented behavior through which employees anticipate and prevent problems, take self-starting action, and actively seek to improve work processes and outcomes (Crant, 2000; Parker et al., 2010). In this study, we conceptualize member task initiative as a behavioral boundary condition that captures between-team differences in members’ proactive task behavior. The social identity account implies that identification must be translated into coordinated task enactment to produce collective performance, and this translation depends on the team’s proactive capacity (Mathieu et al., 2019). When team member task initiative is high, teams are more likely to sustain task execution by proactively addressing emerging problems, improving task processes, and initiating coordination proactively. This is, consistent with the view that initiative involves self-starting, anticipatory efforts to improve work processes and outcomes (Crant, 2000; Parker et al., 2010). Such proactive capacity is consistently associated with stronger team effectiveness, including team productivity and performance outcomes (Lisbona et al., 2020; Lisbona et al., 2021).

Taken together, this study develops an integrative team-level model to clarify when and how leader grandiose narcissism undermines team task performance. Grounded in social identity theory (Ashforth and Mael, 1989; Tajfel and Turner, 1979) and recent evidence on narcissism at the team level (Felty et al., 2015; Grijalva et al., 2020; Yang et al., 2023; Zhou et al., 2019), we argue that leader narcissism reduces team task performance indirectly by eroding team identification and that this indirect effect is contingent on two team member characteristics: member narcissism and member task initiative. This perspective provides a more nuanced understanding of how the dark side of leadership operates at the team level and specifies the conditions under which its effects are likely to be strengthened or weakened.

## 2 Theoretical background and hypotheses

### 2.1 Narcissism

Narcissism was originally introduced by Freud to describe excessive self-love and preoccupation with the self. As personality research has evolved, scholars have increasingly distinguished between narcissistic personality disorder, a clinical diagnosis — and subclinical narcissism, a set of narcissistic tendencies that are continuously distributed in the general population. In organizational behavior and leadership research, the focus is primarily on the latter, treating narcissism as a relatively stable personality trait rather than a psychiatric disorder (Braun, 2017).

Within broader contexts, narcissism is typically discussed alongside Machiavellianism and psychopathy as part of a personality framework termed “the dark triad”. These three traits share low agreeableness and a self-centered orientation, but narcissism is uniquely characterized by a strong self-enhancement motive and a chronic need for external admiration, status, and a sense of superiority (O'Boyle et al., 2015; Paulhus and Williams, 2002). Morf and Rhodewalt (2001) argue that narcissistic individuals simultaneously possess an exaggerated sense of self-importance and entitlement and, at the same time, are heavily dependent on external approval and admiration to stabilize their inherently fragile and unstable self-esteem.

In terms of its manifestations, prior work commonly distinguished between grandiose narcissism and vulnerable narcissism (Miller et al., 2012). Grandiose narcissism is linked to agentic extraversion and is expressed through overt self-confidence, dominance, and a strong need for attention. Vulnerable narcissism, by contrast, builds on the same self-centered antagonism but is accompanied by pronounced neuroticism and insecurity, and is reflected in heightened sensitivity, feelings of hurt, and low self-esteem. Clinical and personality research suggests that narcissism contains both relatively adaptive elements, such as confidence, achievement motivation, and goal orientation, and clearly dark or maladaptive elements, such as entitlement, interpersonal exploitation, and aggressive behavior (Dowgwillo et al., 2016). In organizational and leadership contexts, scholars generally consider grandiose narcissism to be more relevant, as individuals high on this form of narcissism are more inclined to seek power and emerge as leaders (Braun, 2017; Grijalva et al., 2015; O’Reilly and Chatman, 2020). Accordingly, this study focuses on subclinical grandiose narcissism.

At a finer-grained level, the narcissistic admiration and rivalry concept distinguishes admiration and rivalry as two pathways through which grandiose narcissism can be expressed in social contexts (Back, 2018; Back et al., 2013). Although admiration is often linked to relatively more socially functional outcomes and rivalry to more destructive interpersonal outcomes, this distinction should not be treated as equivalent to a general “adaptive versus maladaptive” classification (Back et al., 2013; Dowgwillo et al., 2016). Because this study does not separate these facets empirically in its hypotheses and analyses, it focuses on overall on overall subclinical grandiose narcissism as perceived in leaders and members. To aid readability, Table 1 summarizes the major conceptual distinctions in the narcissism literature and clarifies how they relate to this study.

**Table 1:** Major narcissism-related terms and how they are distinguished.

Distinction (criterion)	Concept 1	Concept 2	Definition / clarification	Representative references
Clinical status	Narcissistic personality disorder (clinical diagnosis)	Subclinical narcissism (trait continuum)	OB/leadership research primarily focuses on subclinical narcissism as a personality trait rather than a psychiatric diagnosis.	Braun (2017)
Manifestation / form	Grandiose narcissism	Vulnerable narcissism	Grandiose narcissism is typically expressed via overt confidence, dominance, and attention seeking, whereas vulnerable narcissism reflects insecurity/hypersensitivity and negative affect. Leadership research generally emphasizes grandiose narcissism in organizational contexts.	Miller et al. (2012) Braun (2017); Grijalva et al. (2015); O’Reilly and Chatman (2020)
Pathways within grandiose narcissism (NARC)	Narcissistic admiration	Narcissistic rivalry	NARC distinguishes two pathways of grandiose expression: admiration (assertive self-promotion/uniqueness striving) and rivalry (defensive self-protection via devaluing others).	Back (2018); Back et al. (2013)
Outcome-oriented lens (not a structural taxonomy)	Relatively more functional outcomes (“adaptive”)	Relatively more destructive outcomes (“maladaptive/dark”)	This is an evaluative lens of outcomes and should not be used interchangeably with structural distinctions such as admiration–rivalry.	Back et al. (2013); Dowgwillo et al. (2016)

Focus of the present study is subclinical grandiose narcissism; admiration and rivalry are discussed for conceptual clarity but are not modeled separately in our analyses. The admiration–rivalry distinction reflects structural pathways, whereas “adaptive versus maladaptive” is an outcome-oriented evaluative lens.

Building on these conceptualizations, unless otherwise specified, narcissism in this study refers to subclinical grandiose narcissism, characterized by exaggerated self-importance, entitlement, and self-centered antagonism. Team leader narcissism is defined as the extent to which team members perceive their formal leader as displaying these grandiose narcissistic tendencies in work settings, whereas member narcissism refers to individual members’ own grandiose narcissistic tendencies. Conceptually, both reflect the same underlying trait; the distinction lies in the target (leader vs. member) and level of analysis in our model. Given our focus on team functioning and performance, our theorizing and empirical tests primarily emphasize the more antagonistic and self-serving expressions of grandiose narcissism, rather than making claims about potentially socially functional expressions.

### 2.2 Team leader narcissism and team task performance

As previously mentioned, narcissistic team leaders typically exhibit strong self-confidence, a sense of authority, and a dominant desire for control (Choi and Phan, 2022). Their excessive pursuit of power may foster a competitive rather than cooperative culture within teams (O’Reilly et al., 2021), leading to poor internal communication and reduced member satisfaction (Mousa et al., 2020), ultimately impairing team task performance. Furthermore, in pursuit of personal achievements, such leaders often prioritize individual goals at the expense of collective team interests (Chen et al., 2023), gradually eroding internal trust and creating communication barriers (Nevicka et al., 2011b; Schmid et al., 2021). Their resistance to feedback and criticism further restricts team-level innovation and adaptability. When team members perceive unfairness and the absence of shared values from their leaders, they may struggle to view their team as a cohesive unit, consequently diminishing their willingness to collaborate and invest effort in team tasks, ultimately impairing overall team performance.

Additionally, this process can be explained by the leader-member exchange theory. Narcissistic leaders tend to establish high quality relationships with team members who enhance their self-image or directly affirm their value (Nevicka et al., 2011b). This behavior creates imbalanced relationships within teams, with some members receiving more attention and resources while others are overlooked. This relational imbalance can weaken overall team cohesion and cooperative spirit, causing members to lose trust because of perceived favoritism and unfairness. Ultimately, morale and engagement decline among those who are inadequately recognized, directly undermining team performance. Taken together, and in line with our conceptualization and measurement of leader narcissism as capturing primarily the antagonistic, entitlement- and exploitation-oriented facets of grandiose narcissism, these arguments suggest that the net effect of leader narcissism on collective task outcomes is predominantly negative. Although grandiose narcissism may sometimes be associated with short-term advantages such as charisma or decisiveness in the early stages of team development, these benefits are likely to be outweighed over time by the relational and coordination costs described above. Accordingly, we hypothesize a negative relationship between leader narcissism and team task performance.

*H1*: Team leader narcissism has a negative effect on team task performance.

### 2.3 Mediating effect of team identification

#### 2.3.1 Team leader narcissism and team identification

Narcissistic leaders are characterized by a heightened sense of self-importance, a strong desire for admiration, and a tendency to make decisions that prioritize their own interests over those of the team (Choi and Phan, 2022; Resick et al., 2009). These behaviors often manifest as dismissing team members’ input and ignoring collective goals, which may foster negative attitudes among subordinates toward both the leader and the organization (Lartey et al., 2024; Zhu et al., 2023).

According to SIT (Ashforth and Mael, 1989), team identification refers to the degree to which individuals perceive themselves as psychologically attached to a team. It involves a sense of emotional commitment and belonging and plays a critical role in shaping team-relevant attitudes and behaviors. Importantly, leaders serve as identity-relevant cues: through what they say and do, leaders shape whether followers perceive them as embodying and advancing “who we are” as a group—that is, as representing “us” and acting as “one of us” (Haslam et al., 2001; Hogg, 2001). From this perspective, leader narcissism should undermine team identification because narcissistic leaders’ self-serving and admiration-seeking behaviors signal weak commitment to collective interests and undermine perceptions that the leader represents and advances the team’s shared identity. When leaders are seen as acting “for themselves” rather than “for us,” the team becomes less attractive as a basis for self-definition and belonging, weakening members’ psychological attachment to the team.

Direct tests linking leader narcissism to team identification remain limited. Nevertheless, evidence across related identification targets and levels provides converging support for the underlying identity-based mechanism that narcissistic leaders can weaken identification with a collective. For example, leader narcissism has been linked to weaker identification-related attachments at other collective levels (e.g., organizational identification) and to team-level identity-relevant bonds with leaders (Li et al., 2025; Wang, 2021). Conceptual work further suggests that leader–member narcissism configurations can trigger subgroup dynamics that undermine collective identification in teams (London, 2019). Together, this body of work supports the plausibility of the identity-based mechanism and highlights the need for a direct test at the team level, as we propose here.

*H2*: Team leader narcissism has a negative effect on team identification.

#### 2.3.2 Team identification mediates the relationship between team leader narcissism and team task performance

Based on the preceding arguments, this study posits that the influence of leader narcissism on team performance is mediated by its effect on team identification. Leader behavior can directly impact team members’ sense of team identification (Bose et al., 2020; Huettermann et al., 2014). According to SIT (Ashforth and Mael, 1989), team identification influences how members perceive their roles within the team and the extent to which they contribute to team goals. Higher team identification is typically associated with greater teamwork, innovative behaviors, and collective contributions (Keem et al., 2023; Song et al., 2018). Research indicates that perceived team identification is often a result of leader behaviors such as thoughtfulness and benevolence (Shaw and Liao, 2021). Team members with a stronger sense of identification with their team are more likely to exhibit proactive work behaviors such as organizational citizenship behaviors (Farmer et al., 2015; Jeong et al., 2025). When team identification is high, employees’ emotions and behaviors shift toward prioritizing the team’s interests over their personal interests (Lin et al., 2016), which enhances team cooperation and performance. Thus, team identification serves as a psychological mechanism that explains how and why leader narcissism may impair team-level outcomes.

*H3*: Team identification mediates the relationship between team leader narcissism and team task performance, such that higher team leader narcissism leads to lower team task performance through reduced team identification.

### 2.4 The moderating effect of employee member narcissism and task initiative

#### 2.4.1 Moderating role of member narcissism in the relationship between team leader narcissism and team identification

Individuals high in narcissism tend to be more self-focused, more concerned with status and external recognition, and more sensitive to cues of relative advantage within the group (Morf and Rhodewalt, 2001). Narcissistic leaders often exaggerate their own contributions, emphasize their superiority, and treat the team as a vehicle for self-display (Grijalva et al., 2015; Rosenthal and Pittinsky, 2006). Under such leadership, teams with a higher average level of member narcissism are more likely to interpret the leader’s narcissistic behaviors as direct competition for status and recognition opportunities rather than merely as role-related behavior. Because narcissistic members are particularly concerned with relative advantage and others’ evaluations, they are more prone to engage in derogating others, competing for dominance, and other dysfunctional conflict behaviors when they perceive the leader as “appropriating” limited attention and resources (Lynch et al., 2022). These processes in turn undermine their psychological attachment to the leader and the team the leader represents. Prior research indicates that narcissism is positively related to provoked aggression in response to ego threat (Rasmussen, 2016) and to higher levels of interpersonal aggression and interpersonal problems in social interactions (Grapsas et al., 2020). This suggests that, in contexts where personal credit is emphasized or “spotlight” is monopolized, highly narcissistic members are more likely to experience dissatisfaction and oppositional reactions.

Recent work further indicates that different combinations of leader and follower narcissism (e.g., high–high vs. high–low) are associated with differential effects on followers’ identification with the leader and creative performance (Liu et al., 2025). These studies focus on dyadic matches between specific leaders and individual followers. This study, by contrast, adopts a compositional perspective and examines team member narcissism as a team-level characteristic. In other words, we are interested in the extent to which teams consist of a greater proportion of highly narcissistic members, and whether such a composition amplifies the detrimental impact of leader narcissism.

From this compositional viewpoint, when the average level of member narcissism in a team is high, the team as a whole becomes more sensitive to perceived status threats and unfairness cues. In such teams, a narcissistic leader’s self-focused behavior and tendency to claim credit are more likely to be interpreted as a direct challenge to members’ self-worth and to the fairness of resource allocation. Members then find it more difficult to view the leader as someone who represents “us,” leading to stronger erosion of team identification. By contrast, in teams with a low overall level of member narcissism, members have weaker needs for personal privilege and distinctiveness and are less inclined to compete with the leader for status. They are therefore more likely to treat a narcissistic leader’s self-focused, credit-claiming behavior as a feature of the role rather than as a personal affront, and not interpreting it as personally abusive or threatening (Nevicka et al., 2018). As a result, when the average level of member narcissism is low, the decline in team identification in response to leader narcissism is likely to be less pronounced.

Based on this reasoning, we expect that when member narcissism is high, the negative relationship between leader narcissism and team identification will be stronger, whereas when member narcissism is low, this negative relationship will be weaker.

*H4*: Member narcissism moderates the relationship between team leader narcissism and team identification such that team leader narcissism has a stronger negative effect on team identification.

#### 2.4.2 Moderating role of member task initiative in the relationship between team identification and team task performance

Task initiative refers to employees’ tendency to spontaneously improve their work methods and processes without direct requests (De Clercq and Mustafa, 2024; Parker et al., 2006). Conceptually, task initiative is a behavioral manifestation rather than a stable personality trait. At the team level, the average level of members’ task initiative reflects an emergent behavioral resource that indicates how proactively a team, as a whole, engages with task-related issues. This initiative not only enhances individual job performance but is also a key factor in boosting team efficacy (Kim et al., 2022; Wihler et al., 2017). According to the self-determination theory, employees with high task initiative can find and execute tasks without external motivation (Strauss and Parker, 2014), which increases intrinsic motivation. Intrinsic motivation is crucial for enhancing job satisfaction and organizational commitment (Aljumah, 2023; Gheitani et al., 2019), both of which are closely associated with employees’ identification with their work team (Marique et al., 2014).

In a team environment, employees with a high level of team identification typically exhibit higher levels of cooperation and greater team synergy (Liao et al., 2022), which directly affect the team’s overall task performance. However, we argue that this positive effect is not purely additive and may depend on the level of task initiative within the team. Rather than conceptualizing task initiative as simply another direct aspect of team effectiveness, we treat the average level of members’ task initiative as a boundary condition that shapes how effectively team identification is translated into performance. Specifically, when team members demonstrate high task initiative, the impact of team identification on task performance may be stronger. Employees with high task initiative actively seek ways to improve their work processes and outcomes. When this initiative aligns with strong team identification, it can lead to enhanced team task performance.

**Figure 1:**
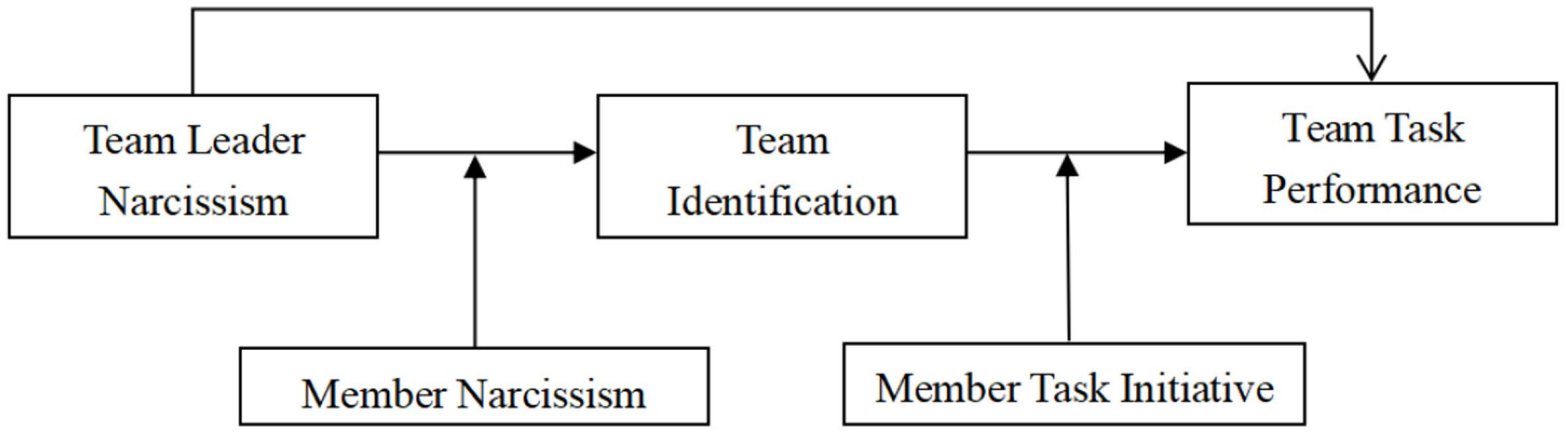
Research model.

*H5*: Member task initiative positively moderates the relationship between team identification and team-task performance such that the positive effect of team identification on task performance is stronger when task initiative is high (Figure 1).

## 3 Methodology

### 3.1 Participants and data collection

The participants were team leaders and members of local enterprises in China representing a range of industries, including manufacturing, services, and information technology. A matched survey design was adopted to enhance measurement validity and reliability. Team members completed questionnaires that assessed leader narcissism, team identification, and demographic variables. Team leaders evaluated their members on narcissism, task initiative, and task performance.

Data were collected by a professional Chinese survey firm. The surveys were distributed and retrieved via WeChat and email. Data were initially collected from 179 work teams, each consisting of one leader and four members, yielding 179 leader responses and 705 member responses. Following a rigorous screening procedure to eliminate insincere and incomplete questionnaires, leader and member surveys were matched within teams. Teams were retained for analysis only when all five questionnaires (one leader and four members) were complete. The final sample consisted of 160 teams, including 160 leaders and 640 team members (800 individuals in total), resulting in a valid response rate of 89%.

To ensure the accuracy of the evaluations, we recorded the tenure of each team member and their direct leader. Considering the results, most members had worked with their leaders for at least 2 years, suggesting sufficient mutual familiarity. More than half (59.5%) of the members had worked with leaders for two to 5 years. The proportion of those who had worked together for less than 1 year was 22%. Members who had worked together for six to 9 years accounted for 17.8%, whereas those who had worked together for more than 10 years accounted for a very low proportion (0.6%). Overall, the tenure patterns suggest moderate-to long-term working relationships between leaders and members, providing a reliable experiential foundation for survey-based assessments.

### 3.2 Measures

All focal constructs in this study were measured using previously validated scales administered via matched leader–member surveys to reduce common method bias. Except for demographic variables, all scale items were rated on a 5-point Likert scale ranging from 1 (strongly disagree) to 5 (strongly agree).

#### 3.2.1 Narcissism

Leader narcissism was assessed using the eight-item adjective-based measure proposed by Resick et al. (2009) and included eight descriptive adjectives. Leader narcissism was measured by aggregating team members’ ratings of their immediate supervisor. Member narcissism was assessed based on supervisors’ evaluations of each subordinate using the same eight-item adjective-based narcissism scale.

We used follower ratings to assess team leader narcissism rather than leader self-reports for both conceptual and methodological reasons. Conceptually, our theorizing focuses on how team members perceive and respond to narcissistic leader behavior, making follower perceptions a theoretically aligned operationalization. Methodologically, prior research has shown that narcissistic individuals tend to engage in self-enhancing response styles and to overestimate their own competence and social desirability, which may undermine the validity of self-reported narcissism, especially in leadership contexts (Judge et al., 2006; Nevicka et al., 2011a). Using follower ratings thus provides a more conservative estimate of leader narcissism and is consistent with recent work on leader narcissism.

#### 3.2.2 Team identification

The scale adapted from Mael and Ashforth (1992) was modified to assess team-level identification by evaluating individuals’ emotional and cognitive attachment to their team. Sample items included statements like “When someone criticizes our team, I feel it as a personal insult” and “When talking about our team, I usually use ‘we’ instead of ‘they’.” Each team member self-rated six items. Individual scores were aggregated to represent team-level identification.

#### 3.2.3 Task initiative

Task initiative was measured by the team leader’s evaluation of each member’s proactive behavior using the three-item scale developed by Griffin et al. (2007). The items included statements such as “Actively pursues more effective ways to perform work tasks” and “Currently exploring ways to improve their working methods.”

#### 3.2.4 Team task performance

Team task performance was assessed based on team leaders’ evaluations of each member’s task execution using a seven-item scale developed by Williams and Anderson (1991). Items included “Able to complete assigned tasks effectively” and “Fulfills job duties and responsibilities well.” Leaders rated each team member on this scale, and we then aggregated these ratings to the team level by computing the mean score for all members within each team, which served as our indicator of team task performance.

#### 3.2.5 Controls variables

We controlled employees’ demographic and relationship-history factors, including gender, age, education level, marital status, employment type, organizational tenure, and years of collaboration with the leader, as well as leaders’ gender, age, education level, marital status, and work experience. These variables were included as control variables to rule out potential demographic- and relationship-history-related confounds (Becker, 2005; Bernerth and Aguinis, 2016).

### 3.3 Research methods

Because employees were nested within teams, we estimated two-level models with individuals (Level 1) nested within teams (Level 2). Our theoretical focus, however, is on team-level processes and team composition. Therefore, individual-level predictors such as member narcissism and member task initiative were decomposed into within-team (Level 1) and between-team (Level 2) components. Consistent with our team-level theorizing, we focus on and report the between-team components as indicators of team composition in the focal structural paths. Accordingly, the effects of these predictors reported in the hypothesis tests should be interpreted as between-team effects rather than purely individual-level effects. Table 2 provides a complete list of all variables and indicates whether each variable was modeled at the within-team level, the between-team level, or decomposed into both components.

**Table 2:** Variable sources, levels of analysis, and component construction.

Variable	Source (rater)	Level in model	Component/construction
Leader narcissism	Members → leader (Resick et al., 2009)	Between (L2)	Aggregated to team mean
Team identification	Members self-report (Mael and Ashforth, 1992)	Between (L2)	Aggregated to team mean
Member narcissism (within)	Leader/supervisor → member (Resick et al., 2009)	Within (L1)	Within-team component (deviation)
Member narcissism (between)	Leader/supervisor → member (Resick et al., 2009)	Between (L2)	Between-team component (team mean; composition)
Task initiative (within)	Leader → member (Griffin et al., 2007)	Within (L1)	Within-team component (deviation)
Task initiative (between)	Leader → member (Griffin et al., 2007)	Between (L2)	Between-team component (team mean; proactive capacity)
Team task performance	Leader → members (Williams and Anderson, 1991)	Between (L2)	Aggregated to team mean

A confirmatory factor analysis (CFA) was conducted to validate the research model’s legitimacy. To test our research hypotheses, we used multilevel modeling of predictor-linked unserved states (MPULS). In this framework, moderation effects are examined by specifying interaction terms between predictors (which may be measured at different levels) to predict the outcome variables. ICC (1), ICC (2), and Rwg are among the most commonly used methods for validating reliability in multilevel organizational research (James et al., 1984). Reliability refers to the consistency of respondents’ answers (Bliese, 2000; Kozlowski and Hattrup, 1992). After confirming reliability and validity, the cross-level effects among the variables were analyzed.

## 4 Research results

### 4.1 CFA

CFA was performed with Mplus 8.3 to evaluate the construct validity of the measurement model. Following standard CFA criteria, factor loadings were expected to be above 0.60, with composite reliability (CR) values exceeding 0.70 and average variance extracted (AVE) surpassing 0.50. As shown in Table 3, the hypothesized five-factor model showed acceptable fit to the data (*χ*^2^ = 673.89, df = 454, CFI = 0.97, TLI = 0.96, RMSEA = 0.06, SRMR = 0.04). To further assess discriminant validity, we compared this five-factor model with several theoretically plausible alternative models that combined conceptually adjacent constructs (Models 2 to 6). The hypothesized five-factor model fit the data better than all alternative models, as reflected in superior fit indices and significant chi-square differences (all *p* < 0.05), supporting the distinctiveness of the focal constructs. Overall, the model fit indices fell within the recommended thresholds, and all measurement indicators met the established criteria, thereby supporting the reliability and validity of the measures.

**Table 3:** Confirmatory factor analysis results for the measurement model.

Model	Factor structure	*χ* ^2^	df	*χ*^2^/df	CFI	TLI	RMSEA	SRMR	Comparison	∆*χ*^2^/df
M1	Hypothesized 5-factor: TLN, TI, MN, MTI, TTP	673.89	454	1.484	0.97	0.96	0.06	0.04		
M2	4-factor: (TLN + MN), TI, MTI, TTP	1064.05	458	2.32	0.91	0.90	0.09	0.08	2 VS 1	390.16 *p* < 0.001
M3	4-factor: TLN, MN, (TI + MTI), TTP	1044.67	458	2.28	0.91	0.90	0.09	0.08	3 VS 1	370.78 *p* < 0.001
M4	3-factor: (TLN + MN), (TI + MTI), TTP	2558.76	461	5.55	0.68	0.65	0.17	0.22	4 VS 3	1514.09 *p* < 0.001
M5	2-factor: (TLN + TI + MN + MTI), TTP	4483.50	463	9.68	0.38	0.34	0.23	0.28	5 VS 4	1924.74 *p* < 0.001
M6	1-factor: all items load on one factor	5451.87	464	11.75	0.23	0.18	0.26	0.31	6 VS 5	968.37 *p* < 0.001

Mxti (Member Task Initiative), MN (Member Narcissism), TTP (Team Task Performance), TLN (Team Leader Narcissism), TI (Team Identification).

### 4.2 Descriptive statistics and correlations

The reliability of the multi-item constructs (member task initiative, member narcissism, team task performance, team leader narcissism, and team identification) was evaluated using Cronbach’s alpha. As shown in Table 4, all Cronbach’s alpha coefficients were above 0.85, indicating satisfactory internal consistency of the scales.

**Table 4:** Descriptive statistics, aggregation indices, and between-team correlations.

Variables	L1 M	L1 SD	ICC1	ICC2	Rwg	L2 M	L2 SD	1	2	3	4	5
Mxti	3.47	1.00	0.54	0.83	0.77	3.47	0.81	(0.90)				
MN	2.82	1.02	0.72	0.91	0.85	2.82	0.91	0.04	(0.93)			
TTP	3.66	0.74	0.76	0.93	0.93	3.66	0.67	0.04	0.02	(0.87)		
TLN	2.21	0.80	0.88	0.97	0.96	2.21	0.76	−0.29***	0.32***	−0.32***	(0.90)	
TI	3.70	0.68	0.66	0.89	0.92	3.70	0.59	0.35***	0.34***	0.33***	−0.23***	(0.91)

The left columns report individual-level descriptive statistics and aggregation indices (ICC1, ICC2, rWG). The correlation matrix is based on team-level (between-team) values. Cronbach’s alpha coefficients are shown on the diagonal in parentheses. ***p < 0.001. Mxti (Member Task Initiative), MN (Member Narcissism), TTP (Team Task Performance), TLN (Team Leader Narcissism), TI (Team Identification).

Relationships among the focal variables were then examined using Pearson correlations based on team means. At the between-team level, team leader narcissism was negatively correlated with both team identification (*r* = −0.23, *p* < 0.001) and team task performance (*r* = −0.32, *p* < 0.001), whereas team identification was positively correlated with team task performance (*r* = 0.33, *p* < 0.001). Member narcissism and member task initiative also showed positive correlations with team identification and team task performance (see Table 4 for full details).

Because employees were nested within teams and our focal relationships were modeled at the team (between) level, we first examined whether the key variables could be meaningfully treated as team constructs or team-composition characteristics. To this end, we calculated intraclass correlations and within-group agreement (Rwg) indices for member task initiative, member narcissism, team task performance, team leader narcissism, and team identification. The ICC(1) values for these variables were 0.54, 0.72, 0.76, 0.88, and 0.66, respectively, all exceeding the commonly accepted threshold of 0.05 (Bliese, 2000), indicating meaningful between-team variance. The corresponding ICC(2) values were 0.83, 0.91, 0.93, 0.97, and 0.89, suggesting high reliability of the between-team components (Klein and Kozlowski, 2000). The Rwg values were 0.77, 0.85, 0.93, 0.96, and 0.92, respectively, further supporting the appropriateness of modeling these constructs at the team level or as team-composition variables (James et al., 1984).

At the team level, Table 4 reports descriptive statistics based on team means for all focal variables. These between-team statistics show that team identification has a mean of 3.70 and a standard deviation of 0.59; team leader narcissism has a mean of 2.21 and a standard deviation of 0.76; team task performance has a mean of 3.66 and a standard deviation of 0.67; member narcissism has a mean of 2.82 and a standard deviation of 0.91; and member task initiative has a mean of 3.47 and a standard deviation of 0.81.

### 4.3 Hypothesis testing

A multilevel linear model is constructed to test the proposed hypotheses. The control variables included employee characteristics (gender, age, education level, marital status, employment type, tenure, and years of collaboration with the leader) and leader characteristics (gender, age, education level, marital status, and work experience). Following prior methodological discussions emphasizing that control variables should be clearly justified and transparently reported (Becker, 2005; Bernerth and Aguinis, 2016), we provide a rationale for the included controls because they may covary with employees’ work attitudes and relationship history, which could otherwise confound the focal relationships. Specifically, age and organizational tenure are naturally correlated and have been linked to job attitudes in prior meta-analytic work (Costanza et al., 2012; Ng and Feldman, 2010). We included employment type because contingent (vs. permanent) employment has been shown to be associated with systematic differences in employees’ work experiences and job attitudes (Wilkin, 2013). We also controlled for marital status as a demographic covariate that has been linked to employees’ job satisfaction and wellbeing in prior research (Peng et al., 2022), and we included it to partial out potential demographic confounds. Finally, we controlled for years of collaboration with the leader because relationship tenure and interaction history can shape dyadic alignment (e.g., LMX agreement), potentially influencing team processes and outcomes (Sin et al., 2009).

In this model, team leader narcissism was the independent variable, team identification was the mediating variable, team task performance was the dependent variable, and member narcissism and member task initiative were the moderating variables.

As shown in Table 5, we estimated four baseline models to examine the main effects of leader narcissism on team identification and team task performance. Model 1 and Model 2 predict team identification, whereas Model 3 and Model 4 predict team task performance. In Models 1 and 3, we entered only the employee- and leader-level control variables. Model 1 predicts team identification from the control variables, with modest explained between-team variance (Between *R*^2^ = 0.03). Model 2 adds team leader narcissism as a focal predictor and shows that team leader narcissism is negatively associated with team identification (*β* = −0.33, *t* = −4.49, *p* < 0.001), increasing Between R^2^ from 0.03 to 0.14.

**Table 5:** Results of team-level regression analysis.

Variable	Team identification				Team task performance			
	Model 1		Model 2		Model 3		Model 4	
	*β*	*t*	*β*	*t*	*β*	*t*	*β*	*t*
Employee gender	0.04	0.88	0.04	0.88	0.01	0.21	0.01	0.21
Employee age	−0.05	−0.70	−0.05	−0.70	−0.08	−1.15	−0.08	−1.15
Employee education	0.02	0.53	0.02	0.53	−0.04	−0.88	−0.04	−0.88
Employee marital status	−0.06	−1.04	−0.06	−1.04	−0.02	−0.43	−0.02	−0.43
Employee form of employment	−0.01	−0.18	−0.01	−0.18	−0.03	−0.74	−0.03	−0.74
Employee years of work	0.00	0.02	0.00	0.02	−0.06	−0.64	−0.06	−0.64
Years working with leader	−0.02	−0.26	−0.02	−0.26	0.09	1.06	0.09	1.06
Leader gender	0.13	1.57	0.13	1.69	0.06	0.71	0.06	0.75
Leader age	−0.08	−0.95	−0.06	−0.67	−0.05	−0.50	−0.03	−0.29
Leader education	−0.06	−0.74	−0.05	−0.59	0.03	0.37	0.04	0.51
Leader marital status	0.03	0.35	0.07	0.90	−0.17*	−2.04	−0.14	−1.70
Leader years of work	0.09	1.07	0.08	0.90	−0.06	−0.65	−0.07	−0.82
Team leader narcissism			−0.33***	−4.49			−0.24**	−3.00
Within *R*^2^	0.00		0.00		0.01		0.01	
Between *R*^2^	0.03		0.14		0.03		0.09	

*N* = 640, † *p* < 0.1, *p < 0.05, **p < 0.01, ***p < 0.001. Dependent variable is Team Identification in Models 1–2 and Team Task Performance in Models 3–4.

Similarly, Model 3 predicts team task performance from the same set of controls (Between *R*^2^ = 0.03), whereas Model 4 adds team leader narcissism and indicates a significant negative association between team leader narcissism and team task performance (*β* = −0.24, *t* = −3.00, *p* < 0.01), increasing Between *R*^2^ from 0.03 to 0.09. Taken together, these results support Hypotheses 1 and 2 and suggest that narcissistic leaders tend to weaken team identification and impair collective performance, consistent with prior research on the detrimental effects of leader narcissism (Choi and Phan, 2022).

To formally test the mediating role of team identification between leader narcissism and team task performance, we estimated the indirect effect using a Monte Carlo simulation approach. Specifically, we used the unstandardized path estimates and their standard errors from the team-level model to generate the sampling distribution of the product term (a × b), where a represents the effect of leader narcissism on team identification and b represents the effect of team identification on team task performance from the model that also included leader narcissism and all control variables. We drew 50,000 random samples from the assumed normal sampling distributions defined by the estimated coefficients and standard errors, computed the product term in each draw, and derived a 95% Monte Carlo confidence interval for the indirect effect. The 95% Monte Carlo CI for the indirect effect was [−0.12, −0.01], which did not include zero.

As shown in Table 6, the results showed that the total effect of leader narcissism on team task performance was significant (total effect = −0.28, SE = 0.07, *t* = −4.26, *p* < 0.001). When team identification was included in the model, the direct effect of leader narcissism on team task performance remained significant but was reduced in magnitude (direct effect = −0.22, SE = 0.07, *t* = −3.32, *p* < 0.001). Most importantly, the Monte Carlo test indicated a significant indirect effect of leader narcissism on team task performance through team identification (indirect effect = −0.06, SE = 0.03, *t* = −2.35, *p* = 0.02).

**Table 6:** Results of mediation analysis.

Path	Effect	S. E.	*t*	*p*	Monte Carlo CI
Total	−0.28	0.07	−4.26	0.00	[−0.41, −0.15]
Direct	−0.22	0.07	−3.32	0.00	[−0.34, −0.09]
Indirect	−0.06	0.03	−2.35	0.02	[−0.12, −0.01]

Taken together, these findings indicate that team identification partially mediates the relationship between leader narcissism and team task performance. Thus, hypothesis 3 was supported.

As shown in Table 7, we estimated four multilevel regression models to examine the main and interactive effects of leader and member narcissism on team identification. Model 1 included only employee and leader-level control variables and showed little explained variance (Within *R*^2^ = 0.01, Between *R*^2^ = 0.03). Model 2 added team leader narcissism as a predictor; team leader narcissism had a significant negative effect on team identification (*β* = −0.24, *t* = −3.00, *p* < 0.01). In Model 3, we further entered member narcissism, which was positively related to team identification (*β* = 0.29, *t* = 8.02, *p* < 0.001), while the negative effect of team leader narcissism remained significant (*β* = −0.24, *t* = −3.00, *p* < 0.01). Model 4 then included the interaction term team leader narcissism × member narcissism to test the proposed moderation effect. The results showed that the interaction between leader narcissism and member narcissism in predicting team identification was significant (*β* = −0.12, *t* = −3.25, *p* < 0.01), supporting hypothesis 4.

**Table 7:** Results of regression analysis for hypothesis 4.

Variable	Team identification							
	Model 1		Model 2		Model 3		Model 4	
	*β*	*t*	*β*	*t*	*β*	*t*	*β*	*t*
Employee gender	0.01	0.21	0.01	0.21	0.03	0.66	0.02	0.59
Employee age	−0.08	−1.15	−0.08	−1.15	−0.08	−1.22	−0.10	−1.56
Employee education	−0.04	−0.88	−0.04	−0.88	−0.03	−0.79	−0.04	−0.89
Employee marital status	−0.02	−0.43	−0.02	−0.43	−0.05	−0.89	−0.05	−0.95
Employee form of employment	−0.03	−0.74	−0.03	−0.74	−0.02	−0.61	−0.03	−0.68
Employee years of work	−0.06	−0.64	−0.06	−0.64	−0.07	−0.76	−0.06	−0.75
Years working with leader	0.09	1.06	0.09	1.06	0.09	1.17	0.11	1.43
Leader gender	0.06	0.71	0.06	0.75	0.06	0.75	0.06	0.75
Leader age	−0.05	−0.50	−0.03	−0.29	−0.03	−0.29	−0.03	−0.29
Leader education	0.03	0.37	0.04	0.51	0.04	0.51	0.04	0.51
Leader marital status	−0.17*	−2.04	−0.14	−1.70	−0.14	−1.70	−0.14	−1.70
Leader years of work	−0.06	−0.65	−0.07	−0.82	−0.07	−0.82	−0.07	−0.82
Team leader narcissism			−0.24**	−3.00	−0.24**	−3.00	−0.24***	−3.00
Member narcissism					0.29***	8.02	0.28***	7.46
Team leader narcissism * member narcissism							−0.12**	−3.25
Within *R*^2^	0.01		0.01		0.09		0.11	
Between *R*^2^	0.03		0.09		0.09		0.09	

*N* = 640, †*p* < 0.1, *p < 0.05, **p < 0.01, ***p < 0.001.

**Figure 2:**
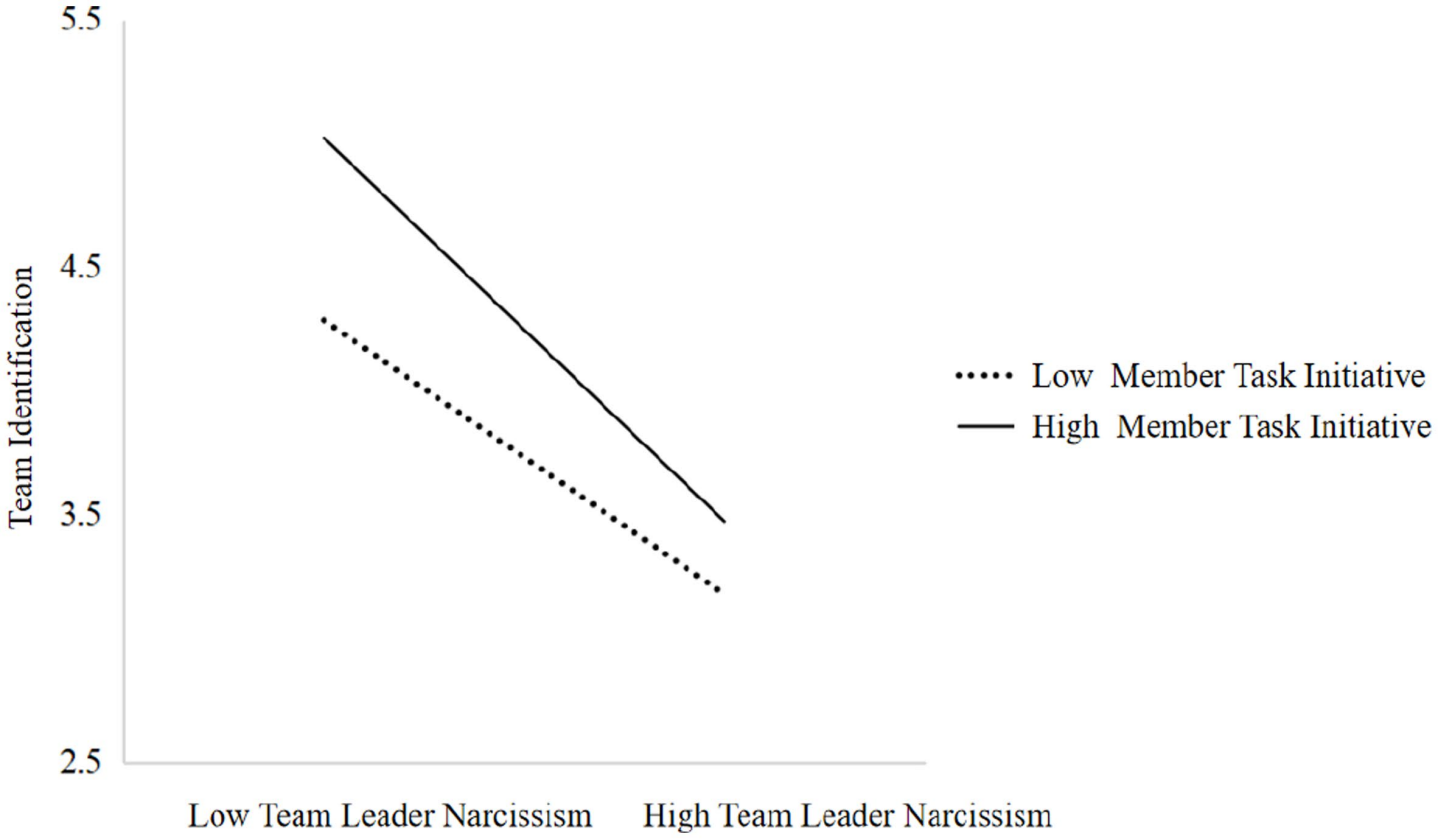
Moderating effect of member narcissism.

To understand the nature of moderation, a simple slope test was conducted. The results, depicted in Figure 2, show that when member narcissism is high, the relationship between leader narcissism and team identification is strong (+1 SD; *β* = −0.36, *p* < 0.001), but when member narcissism is Low, the relationship weakens (−1 SD; *β* = −0.12, *p* > 0.05). In other words, the negative effect of leader narcissism on team identification was more pronounced when team members exhibited higher levels of narcissism. One possible explanation is that highly narcissistic members tend to resist controlling and competitive leadership behaviors and are less likely to accept authority, thereby further weakening their identification with the team.

As shown in Table 8, the interaction between team identification and member task initiative (i.e., the average level of member task initiative within each team, corresponding to the between-team component) had a significant positive effect on team task performance. Model 1 included only employee- and leader-level control variables (Within *R*^2^ = 0.00, Between *R*^2^ = 0.03). Consistent with our baseline model, Model 2 first added team leader narcissism as a focal predictor of team task performance, which showed a significant negative association with team task performance (*β* = −0.33, *t* = −4.49, *p* < 0.001). Model 3 further included team identification, which was positively related to team task performance (*β* = 0.32, *t* = 4.08, *p* < 0.001), while team leader narcissism remained significant (*β* = −0.25, *t* = −3.39, *p* < 0.001; Between *R*^2^ = 0.23). Model 4 added the main effect of team-level member task initiative, which was not significant (*β* = 0.04, *t* = 0.92). To test the proposed moderation, Model 5 added the interaction term team identification × member task initiative, which was significant and positive (*β* = 0.12, *t* = 2.87, *p* < 0.001), supporting hypothesis 5.

**Table 8:** Results of regression analysis for hypothesis 5.

Variable	Team task performance									
	Model 1		Model 2		Model 3		Model 4		Model 5	
	*β*	*t*	*β*	*t*	*β*	*t*	*β*	*t*	*β*	*t*
Employee gender	0.04	0.88	0.04	0.88	0.04	0.88	0.04	0.92	0.03	0.87
Employee age	−0.05	−0.70	−0.05	−0.70	−0.05	−0.70	−0.05	−0.71	−0.05	−0.79
Employee education	0.02	0.53	0.02	0.53	0.02	0.53	0.02	0.57	0.02	0.49
Employee marital status	−0.06	−1.04	−0.06	−1.04	−0.06	−1.04	−0.06	−1.02	−0.06	−1.17
Employee form of employment	−0.01	−0.18	−0.01	−0.18	−0.01	−0.18	−0.01	−0.19	−0.01	−0.28
Employee years of work	0.00	0.02	0.00	0.02	0.00	0.02	0.01	0.07	0.01	0.13
Years working with leader	−0.02	−0.26	−0.02	−0.26	−0.02	−0.26	−0.02	−0.29	−0.04	−0.48
Leader gender	0.13	1.57	0.13	1.69	0.11	1.49	0.11	1.50	0.11	1.50
Leader age	−0.08	−0.95	−0.06	−0.67	−0.05	−0.60	−0.05	−0.60	−0.05	−0.60
Leader education	−0.06	−0.74	−0.05	−0.59	−0.06	−0.80	−0.06	−0.80	−0.06	−0.80
Leader marital status	0.03	0.35	0.07	0.90	0.12	1.52	0.12	1.52	0.12	1.52
Leader years of work	0.09	1.07	0.08	0.90	0.10	1.23	0.10	1.23	0.10	1.23
Team leader narcissism			−0.33***	−4.49	−0.25***	−3.39	−0.26***	−3.41	−0.25***	−3.41
Team identification					0.32***	4.08	0.32***	4.11	0.32***	4.13
Member task initiative							0.04	0.92	0.01	0.15
Team identification * member task initiative									0.12***	2.87
Within *R*^2^	0.00		0.00		0.00		0.01		0.02	
Between *R*^2^	0.03		0.14		0.23		0.23		0.23	

*N* = 640, †*p* < 0.1, *p < 0.05, **p < 0.01, ***p < 0.001.

**Figure 3:**
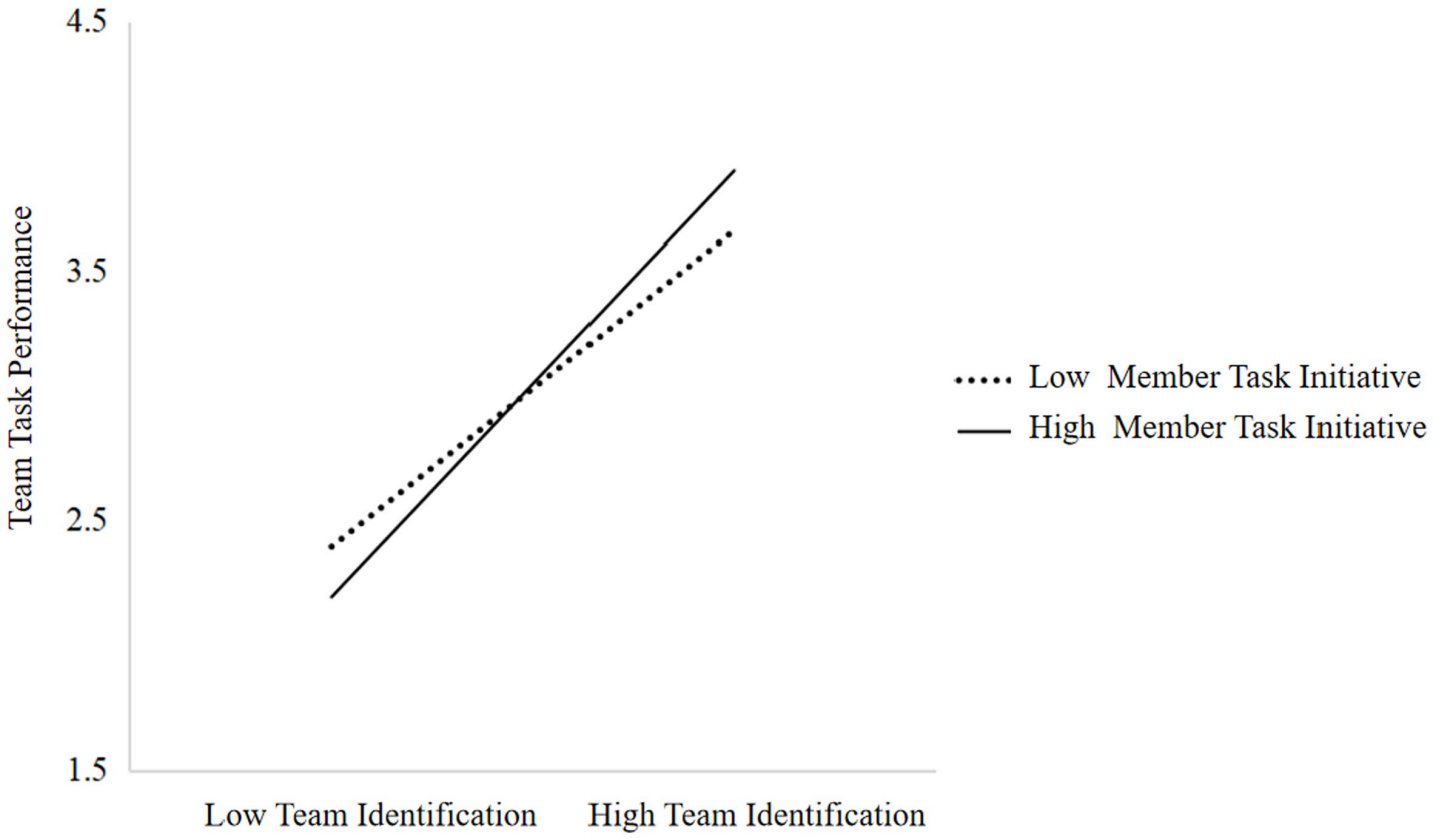
Moderating effect of member task initiative.

To further interpret this moderating effect, we conducted a simple slope analysis at the between-team level. As depicted in Figure 3, when team-level member task initiative was high (+1 SD), the conditional effect of team identification on team task performance was stronger (*β* = 0.44, *p* < 0.001), whereas when team-level member task initiative was low (−1 SD), this effect was weaker (*β* = 0.20, *p* < 0.05). These results indicate that higher average task initiative strengthens the positive association.

## 5 Discussion

This study provides a comprehensive analysis of the impact of leader narcissism on team task performance, specifically examining the mediating role of team identification and the moderating effects of subordinate narcissism and task initiative. Using multilevel data and team-level analyses, this study shows that leader narcissism is negatively associated with team task performance and that this relationship is partially mediated by lower team identification. Subordinate narcissism exacerbates the negative effects of leader narcissism, whereas task initiative significantly influences the relationship between team identification and task performance.

In line with prior work portraying narcissism as a “double-edged sword,” we also acknowledge that leader narcissism may yield short-term or context-dependent benefits, for example, when its agentic, confident, and bold decision-making aspects help mobilize teams around ambitious goals. Even though leader narcissism may yield short-term or context-dependent benefits, our findings in a team-based context highlight its predominantly negative, identity-related consequences. Specifically, leader narcissism negatively correlates with team task performance, with team identification partially mediating this effect. Narcissistic leaders, characterized by inflated self-confidence, dominance, and neglect of subordinates’ perspectives, undermine team cohesion and reduce members’ collective belonging, negatively impacting coordination and overall team performance. These results align with those of previous studies, such as Araujo-Cabrera et al. (2017) and Leblanc et al. (2022), which highlight how leaders’ personality traits influence team structures and outcomes. Additionally, Wang et al. (2021) examined the relationship between narcissistic supervision and employees’ change-oriented OCBs. Collectively, this body of work supports the notion that narcissistic leaders negatively affect team performance through variables such as employee psychology or commitment.

Moreover, member narcissism significantly exacerbates the relationship between leader narcissism and team identification. Specifically, higher member narcissism magnifies the adverse effects of leader narcissism on team identification, as narcissistic team members may be particularly sensitive to authoritarian, self-centered leadership, reducing their psychological attachment to the team. Conversely, teams with lower member narcissism are less negatively affected by leader narcissism and tend to maintain relatively stable levels of team identification. These findings underline the importance of congruence or incongruence between leader and follower personalities, enhancing the understanding of person-team fit and identity processes.

In addition to this moderating pattern, our analyses also revealed a direct positive relationship between member narcissism and team task performance, even though this effect was not part of our core hypotheses. This result suggests that, in task-focused teams, narcissistic members’ strong concern for status and performance can, under certain conditions, be converted into greater effort and more visible contributions to team outcomes. Prior studies have similarly shown that the “agentic” or admiration-related facet of narcissism is associated with status striving, achievement-oriented behavior, and improved performance when opportunities for recognition are salient (Zeigler-Hill et al., 2019). In this sense, while a higher average level of member narcissism may weaken team identification in the presence of leader narcissism, it may at the same time be linked to better task performance because narcissistic members are highly motivated to display competence and surpass others (Furnham and Treglown, 2021; Zhao et al., 2023). Taken together, these findings portray member narcissism as a “double-edged sword”: it may bring performance-related benefits, but at the cost of relational strain and identity-related vulnerabilities. We view this unexpected positive association between member narcissism and team performance as a worthwhile topic for future research, particularly in exploring when and how narcissistic tendencies can be channeled toward task accomplishment without eroding team cohesion.

Third, member task initiative moderates the relationship between team identification and team task performance. The regression analysis reveals a significant positive interaction between team identification and member task initiative, suggesting that task initiative generally amplifies the positive effect of identification on performance. This pattern is consistent with prior research on proactive work behavior, which suggests that self-starting, change oriented action enables teams to convert shared identification and motivation into tangible performance gains (Strauss and Parker, 2014).

### 5.1 Theoretical implications

Contrary to clear claims of a widespread “narcissism epidemic,” recent longitudinal studies and meta-analyses revealed that narcissism among younger cohorts either stabilized or declined over the past decades (Oberleiter et al., 2025). For example, Twenge et al. (2021) reanalyzed data from U. S. college students spanning 1982–2016 and found a significant decline in narcissism following the Great Recession, suggesting a societal shift toward humility, cooperation, and subjective well-being. Similarly, cross-cultural analyses by Oberleiter et al. (2025) confirmed that grandiose narcissism levels generally decreased while communal values and interpersonal warmth gained importance. These findings challenge the narrative of ever-increasing narcissism (Twenge et al., 2008; Twenge et al., 2021) and suggest that, in contrast with previous concerns, dysfunctional interpersonal interactions stemming from narcissistic traits among team members may be less prevalent, thereby potentially reducing interpersonal conflict and enhancing collaboration within modern teams.

Nevertheless, narcissism among leaders remains visible and influential. Narcissistic leaders frequently emerge in organizational settings because of the behavioral dynamics associated with grandiose narcissism, such as charisma, self-assuredness, and dominance (Back et al., 2013; Krizan and Herlache, 2018). According to self-regulatory models, narcissists actively seek positions of power and recognition, such as leadership roles, to maintain and enhance their inflated self-concepts through external validation and admiration (Morf and Rhodewalt, 2001; Wallace and Baumeister, 2002). Leadership roles provide ideal platforms for self-promotion and displays of competence and superiority (Campbell and Campbell, 2009).

This study expands on existing research on leader narcissism by emphasizing the mediating role of team identification in the relationship between leader narcissism and team performance. These findings confirm and expand on prior research (Wilhau, 2021), illustrating how leader narcissism disrupts team cohesion and reduces team performance. Furthermore, incorporating subordinate narcissism and task initiative as moderators enriches the understanding of individual differences that influence the team dynamics created by leader narcissism. Notably, this study addresses a critical gap concerning the effects of narcissism on team task performance. While prior studies predominantly examined direct effects or leader-subordinate interactions focused on innovation, this research broadens the scope by explicitly integrating subordinate narcissism as a moderator. Investigating the combined impact of leader and subordinate narcissism on team identification clarifies the dyadic and collective mechanisms underlying team processes.

Leveraging SIT (Ashforth and Mael, 1989; Tajfel, 1974), this study demonstrates leader narcissism’s significant detrimental effect on team identification, subsequently impairing performance. Subordinates’ narcissism further intensifies this path; when members exhibited higher narcissistic tendencies, the detrimental effect of leader narcissism on team identification became more pronounced, thereby amplifying its negative indirect impact on team performance. Additionally, empirically confirming the moderating role of task initiative expands the application of the self-determination theory within team contexts, illustrating how intrinsic motivation arising from proactive task engagement interacts intricately with identification and leadership dynamics. These insights underscore the double-edged nature of task initiative in highly identified teams.

### 5.2 Practical implications

This study underscores the importance of careful selection and management of leaders with narcissistic tendencies. Organizations can invest in leadership development that strengthens empathy, emotional intelligence, and team-building skills to reduce the potential downsides of leader narcissism. Moreover, accounting for subordinate characteristics may help managers deploy more effective team strategies when working with narcissistic leaders.

Specifically, during leader selection, organizations should be mindful of narcissism-related risks and adopt comprehensive assessment procedures, including psychological assessments, behavioral interviews, and 360-degree evaluations, to effectively identify suitable leadership candidates. For current leaders, tailored training programs that address narcissistic behaviors and promote team identification and cooperation can encourage more inclusive and effective leadership styles.

Based on our findings, we recommend that organizational managers pay close attention to the combined effects of leader and member narcissism within teams, particularly in team environments characterized by high levels of collaboration and group identification. Specifically, managers can mitigate internal conflicts and the erosion of team identification caused by power and status competition by reducing the overall narcissistic climate within teams, thus enhancing team cohesion and organizational effectiveness. Furthermore, when team identification is negatively affected by leader narcissism, organizations should proactively foster members’ task initiative to compensate for potential declines in team performance. Such interventions can be realized by establishing clear team goals and appropriate incentive mechanisms to strengthen members’ intrinsic motivations and autonomous engagement. Enhancing members’ task initiative may effectively alleviate the detrimental influence of narcissistic leaders on team morale and enable better adaptation and performance under complex conditions.

Finally, owing to the crucial mediating role of team identification, organizations should cultivate an inclusive, respectful, and supportive workplace culture. Developing such an environment enhances interpersonal trust, collaboration, and team identification, thereby safeguarding against the negative effects of narcissistic leaders. This cultural transformation can substantially enhance team collaboration and organizational performance, thus reducing the hidden costs associated with toxic leadership behavior. From a managerial perspective, carefully monitoring narcissistic traits, providing humility-oriented training, strategically managing team personality composition, and balancing task initiatives can mitigate adverse outcomes and optimize team performance.

### 5.3 Limitations and directions for future research

This study offers valuable contributions to the understanding of how leader narcissism influences team identification and task performance, particularly by illuminating the moderating roles of subordinate narcissism and task initiative. However, several limitations must be acknowledged, which also provide meaningful directions for future research.

First, reliance on cross-sectional data limits the capacity to establish causal inferences. Without longitudinal evidence, it remains uncertain whether these observed relationships are causal. It remains unclear whether the effects of leader narcissism on team dynamics and outcomes persist over time or fluctuate across the different developmental stages of team functioning. Future research should employ longitudinal or multiwave designs to capture the temporal dynamics and causal mechanisms underlying the relationships identified in this study. Additionally, although we relied on multilevel (nested) data, our focal model was estimated at the between-team level, treating member narcissism and task initiative as team-composition or emergent characteristics. Future studies could complement this approach with genuinely cross-level designs, such as linking leader narcissism at the team level to individual level identification, well-being, and performance, or testing more fine-grained leader follower narcissism (in)congruence patterns. Such work would help to build a more comprehensive picture of how different forms and configurations of narcissism unfold across levels in teams and organizations.

Second, generalizability of the findings may be limited by the sample characteristics. Data were collected from a specific cultural and industrial context, which may not reflect variations in leadership behaviors and team dynamics across different cultural or organizational environments. Future studies should replicate and extend these findings across diverse cultural settings and industries to assess the boundary conditions of the observed effects.

Third, although this study included subordinate narcissism and task initiative as moderators, other potentially important moderators and mediators were not examined. Factors such as perceived organizational support, job satisfaction, and psychological safety can also influence the impact of leader narcissism on team functioning. Incorporating these variables in future research will provide a more comprehensive understanding of the mechanisms and contingencies at play.

Moreover, this study primarily focuses on leader narcissism as a single dark trait. Future research should consider a broader array of dark personality characteristics, particularly those comprising the “Dark Triad” (narcissism, Machiavellianism, and psychopathy), and examine how these traits co-occur and interact to shape toxic leadership and team outcomes. At the same time, we modeled leader narcissism as a unidimensional, grandiose and maladaptive construct at the team level and did not distinguish between different narcissism facets (e.g., admiration vs. rivalry) or profile combinations of leader and member narcissism. Building more directly on the “double-edged” view of narcissism, future studies could adopt multidimensional measures that differentiate admiration from rivalry and investigate whether the patterns observed here differ across more agentic/admiration-related versus more antagonistic/rivalry-related expressions of leader narcissism, as well as across different combinations of leader and follower narcissism. Such work would provide a more nuanced picture of when and how distinct dark traits and different narcissism profiles jointly influence team identification, team dynamics, and performance.

By addressing these limitations, future research can build a more robust theoretical and empirical foundation for understanding the multifaceted effects of leader narcissism and related traits within organizational contexts.

## Data Availability

The datasets presented in this study can be found in online repositories. The names of the repository/repositories and accession number(s) can be found at: https://osf.io/t5xyg/overview.
