# Correction: How team leader narcissism influences team task performance: the mechanisms of team identification, team member narcissism and task initiative

**DOI:** 10.3389/fpsyg.2026.1822166

**Published:** 2026-03-13

**Authors:** Frontiers Production Office

**Affiliations:** Frontiers Media SA, Lausanne, Switzerland

**Keywords:** social identity theory, team identification, team leader narcissism, team member narcissism, team task performance, team member task initiative

An incorrect **Funding** statement was provided. The correct **Funding** statement reads:

“The author(s) declared that financial support was received for this work and/or its publication. This research was funded by Dong-A University.”

The original version of this article has been updated.

